# Blending of rodenticide and battery acid – a rare and fatal suicide mix

**DOI:** 10.1186/s41935-017-0002-1

**Published:** 2017-07-18

**Authors:** Nisreen Abdul Rahman, Siddhartha Das, Vinod Ashok Chaudhari, Suresh Nandagopal, Bhawana Badhe

**Affiliations:** 1 Department of Forensic Medicine & Toxicology, JIPMER, Puducherry, India; 2 Toxicology division, Forensic Sciences Department, Chennai, India; 3 Department of Pathology, JIPMER, Puducherry, India

**Keywords:** Poisoning, Rodenticide, Aluminium phosphide, Battery acid, Chemical reaction, Autopsy findings

## Abstract

**Background:**

Self-poisoning usually occurs by the ingestion of a single lethal compound and majority of the poisoning cases in our country are due to the pesticides. Suicide by ingestion of more than one poisonous substance is rare except alcohol and multiple drugs. To the best of our knowledge, suicidal ingestion of a mixture of rodenticide and battery acid has not been reported before in medical literature.

**Case presentation:**

Here we are discussing a case of simultaneous ingestion of two poisonous substances, i.e., aluminium phosphide and battery acid. In general, an acid is mainly locally acting while metal phosphide is a systemic poison. Features suggestive of both these poisonous substances were noticed on clinical, autopsy and histopathological examination and supported by a positive chemical analysis report of viscera. Moreover, we analysed the possible reactions which may occur between these two compounds and their by-products outside and inside our body.

**Conclusion:**

In cases where, combination of poisons is suspected a proper history, meticulous autopsy and ancillary investigation including histopathogy and analytical toxicology are required to conclude the cause of death and mechanism of poisoning.

## Background

As per National Crime Records Bureau (NCRB) statistics, about 1,31,666 people lost their lives by committing suicide during the year 2014 in India. Poisoning was observed to be the second most common method of suicide (National Crime Records Bureau 2015). Reports showed that the rate of poisoning in developing countries ranges from 0.07 to 0.7% (Zöhre et al. 2015). The compound used for poisoning in a particular area depends largely upon the predominant occupation of the area, agricultural settings and availability of various substances under the existing law (Pal et al. 2011). According to Srivastava et al. highest incidence of poisoning was due to household agents (44.1%) which include pyrethroids, rodenticides, carbamates, phenyl, detergents and corrosives (Srivastava et al. 2005; Singh & Kaur 2012). Usually single poison is used by the victim to kill himself. Rarely a combination of poisonous substances other than alcohol and multiple drugs are used (Zöhre et al. 2015). In this case report we are discussing an unusual case of fatal poisoning due to ingestion of a mixture of rodenticide and a mineral acid. We will be analysing the possible interactions between these two poisons and their effects on the body.

## Case presentation

An automobile mechanic with an alleged history of consumption of rat killer poison mixed with battery acid presented with coffee coloured vomitus from the nasal and oral cavities. Doctors treated him with cold saline stomach wash, antibiotics and other supportive measures (i.v fluids, vitamin K, oxygen inhalation etc.). The blood investigations showed raised liver transaminases (SGOT: 208 IU/L, SGPT: 228 IU/L), serum urea (177 mg/dl), serum creatinine (3.6 mg/dl), PT (18.7 s), INR (1.5) and random blood sugar (RBS: 309 mg/dl). The next day he had a decrease in haemoglobin level from 11.7 to 8 g/dl and increase in fasting blood sugar (FBS: 280 mg/dl). The values of SGOT (268 IU/L), SGPT (300 IU/L), PT (22.5 s), INR (2.4), urea (221 mg/dl), creatinine (4 mg/dl) also increased. Clinical examination at our hospital revealed congestion and ulceration of the oral cavity and posterior pharyngeal wall and, tenderness over the epigastric region.

Silver nitrate test (a filter paper impregnated with silver nitrate is placed in the mouth of the patient to breathe through it for 15–20 min, blackish discolouration of the paper indicates presence of phosphine in breath) which aims to detect the exhaled phosphine gas from breath was positive at the time of admission. Arterial blood gas analysis revealed metabolic acidosis (blood pH-7.2, HCO_3_^−^-15 mEq/L, paCO_2_-30 mmHg). He suddenly developed breathlessness and treating physician advised CT scan of thorax. It showed moderate pleural effusion on the left side, posteromedial basal segment collapse and bilateral perihilar ground glassing of upper and lower lobes. These features were suggestive of diffuse alveolar damage. CT abdomen showed mild ascites. Subsequently, he developed features of renal failure (serum urea: 66 mg/dl, serum creatinine: 3.3 mg/dl), disseminated intravascular coagulation (prothrombin time: 22.5 s, INR: 2.4) and expired on the fifth day.

**Fig. 1:**
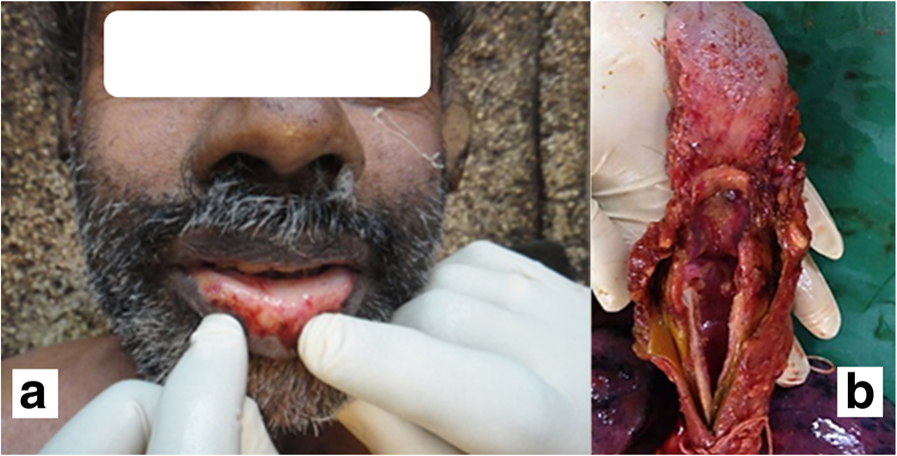
**a** Erosion and haemorrhages over the lip. **b** Erosion and haemorrhages over the tongue

**Fig. 2:**
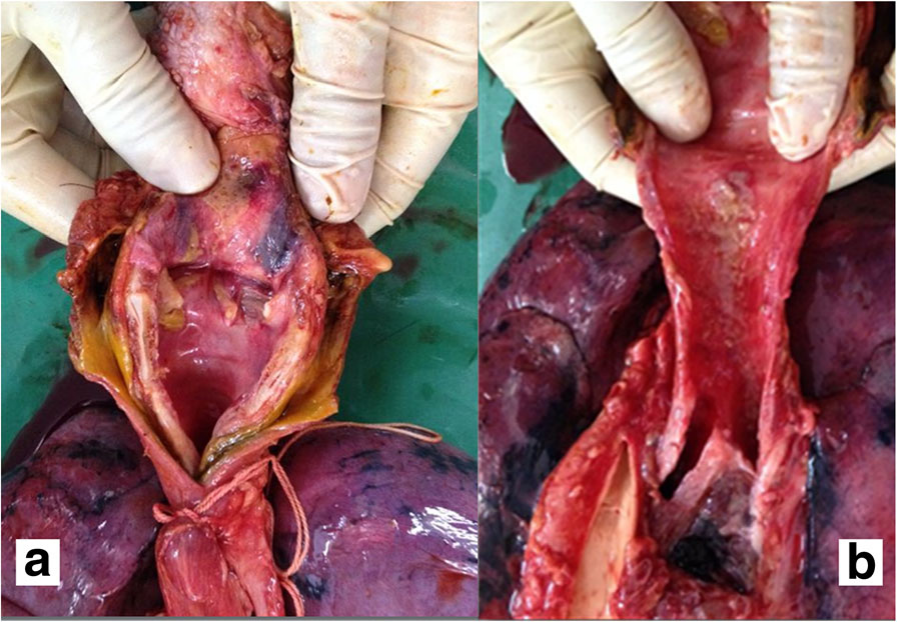
**a** Haemorrhage and yellowish discolouration of mucosa in the glottic region. **b** Mucosal corrosion with yellowish plaques in the larynx, trachea and bronchus

**Fig. 3:**
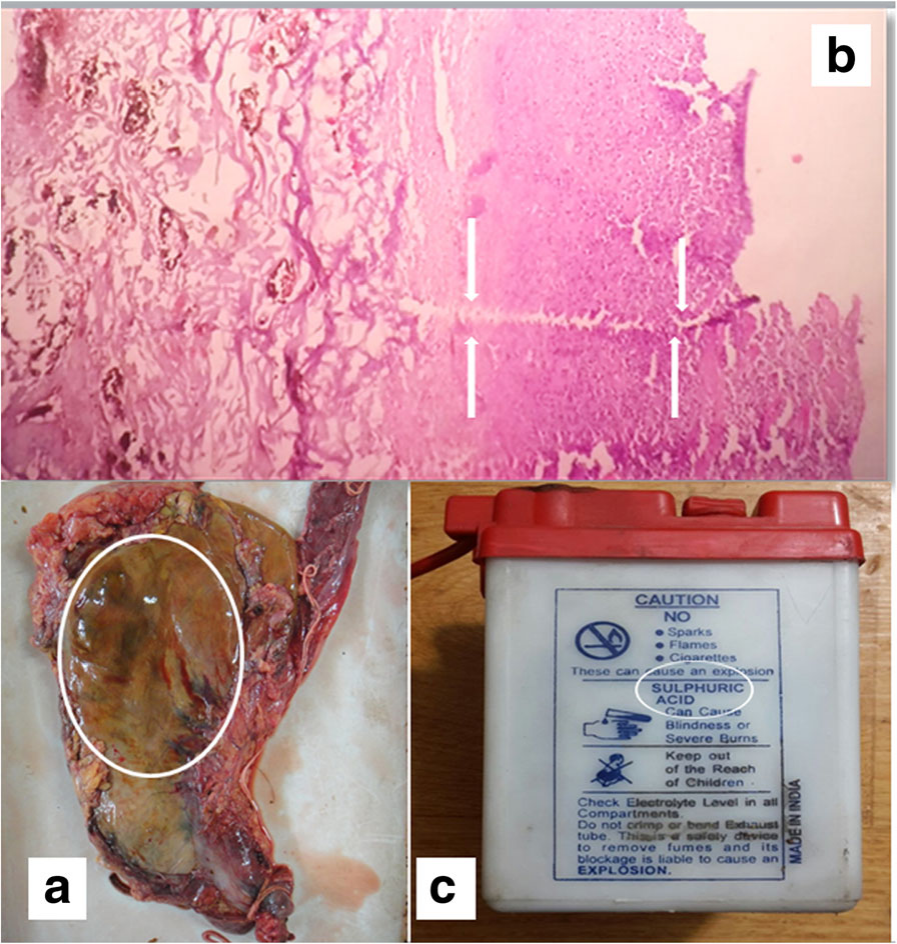
**a** Thinning of posterior wall of the stomach. **b** Histopathology showing transmural necrosis of the stomach wall (H & E staining–100x). **c** The battery acid used for the purpose showing sulphuric acid as the constituent

**Fig. 4:**
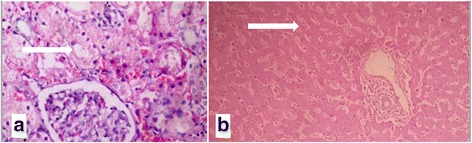
**a** Acute tubular necrosis of kidney (H & E staining–400x). **b** Sinusoidal congestion of liver (H & E staining–100x)

At autopsy erosions and minute haemorrhages were seen on both lips and dorsal aspect of the tongue (Fig. 1). Internal examination showed mucosal corrosion with yellowish plaques over epiglottis, glottis, trachea and bronchi (Fig. 2). Both pleural cavities contained 200 ml of exudate. Both the lungs showed congestion and oedema. Oesophagus showed yellowish brown coloured coating, with oedema of the wall. Peritoneal cavity contained 300 ml of exudate. The stomach contained 200 ml of dark brown coloured fluid and the mucosa coated blackish brown in colour with thinning of the posterior surface near the greater curvature (Fig. 3). Liver, kidney and the intestinal walls showed petechial haemorrhages. Histopathology examination by using haematoxylin and eosin staining showed diffuse acute tubular necrosis of kidney, sinusoidal congestion of the liver, and transmural necrosis of stomach wall (Figs. 3 & 4). The chemical analysis report of viscera detected sulphate ions and aluminium probably derived from a sulfuric acid and aluminium phosphide respectively.

## Discussion

The commonest agent of poisoning in rural India is aluminium phosphide (AlP), as compared to organophosphorus compounds in other areas (Singh & Kaur 2012). It is easily available in India and sold under trade names like celphos, quickphos, etc. as tablets or pellets. A tablet weighs 3 g containing 56% of AlP and 44% of ammonium carbonate to reduce self-ignition (Aggrawal 2006). Phosphine gas is produced from this on reaction with moisture or acid. It acts as a mitochondrial poison by inhibiting cytochrome C oxidase and leads to reduced oxidative phosphorylation, ATP depletion and cell death (Pal et al. 2011; Singh et al. 2014; Soltani et al. 2013). It has toxic effects on all the systems and induces acid–base disturbances (Soltani et al. 2013). Moreover, phosphides and phosphine have a corrosive action (Pal et al. 2011). The type of acid, quantity, physical structure, pH, concentration and its affinity for hydroxyl ions are responsible for the severity of tissue injury from an acid (Pillay 2013; Contini & Scarpignato 2013). The primary mechanism of action of mineral acid is the coagulation necrosis of tissues. Battery acid contains about 30% of sulfuric acid (Fig. 3), which produces local effect and on systemic absorption there can be metabolic acidosis, haemolysis and acute renal failure (Pillay 2013; Wormald & Wilson 1993). The deceased being a mechanic in an automobile shop had easy access to battery acid while rodenticides are available at a cheap rate in the market. These could be a facilitator for a method of suicide in this case. Fatal poisoning by a combination of these two compounds has not been reported in medical literature.

According to Singh Y et al. (Singh et al. 2014) AlP poisoning showed features of vomiting, abdominal pain, acidosis, marked hypotension, palpitation, acute renal failure, acute liver failure, disseminated intravascular coagulation (DIC) clinically. Phosphine gas causes injury to the alveolar capillary membrane and acute lung injury (Gurjar et al. 2011). This gas on absorption to blood stream damages the blood vessel and RBC cell membrane causing intravascular haemolysis (Pal et al. 2011). It may be the possible reason for reduced haemoglobin level in the present case. He was non-diabetic but had hyperglycaemia and the level of which is one of the prognostic sign of mortality (Mehrpour et al. 2008). Moreover, he had features of abdominal pain, vomiting, acute renal and liver failure, diffuse alveolar damage and DIC suggestive of phosphide poisoning. The effusion in the pleural cavity and peritoneum could be due to liver and kidney failure.

Sulfuric acid has a great affinity for water and produces a severe rise in temperature on reaction with it to cause eschar. Ingestion of this acid causes burning pain from oral cavity to stomach, hematemesis (coffee coloured vomiting), excessive salivation, dysphagia, dysphonia and dyspnoea. (Pillay 2013) It causes more coagulative necrosis of columnar mucosa of the stomach than to the stratified squamous epithelium of the oesophagus (Arévalo-Silva et al. 2006). But as per one study, oesophageal corrosions were observed in 55% of battery acid ingestion cases (Wormald & Wilson 1993). He had coffee coloured hematemesis which is one of the presenting features of an acid poisoning. Abdominal pain, dyspnoea and vomiting are common to both poisons, and we also observed in the instant case.

The autopsy revealed features of acid poisoning more distinctively. The stomach mucosa showed blackish brown discolouration and thinning of the wall; suggestive of damages caused by sulfuric acid. In a death due to acid ingestion; autopsy showed distal third oesophagus perforation, stomach corrosion, pulmonary and cerebral oedema (Amadasi et al. 2016). We also, observed similar autopsy findings.

Atypical findings noticed in our case may be the result of two poisonous chemical substances which reacted with each other both inside and outside the body. The combination has a unique peculiarity that, when AlP comes in contact with acid there will be more extraction of phosphine gas as given by equation below (Chan et al. 1983; Proudfoot 2009; De 2007).

$$ 2\ \mathrm{A}\mathrm{l}\mathrm{P} + 3\ {\mathrm{H}}_2{\mathrm{SO}}_4\to {\mathrm{Al}}_2{\left({\mathrm{SO}}_4\right)}_3 + 2\ {\mathrm{PH}}_3 $$

It could be the probable reason for not finding any typical features of sulfuric acid ingestion inside the oral cavity or larynx. The same principle used for the enhanced detection of phosphine gas from post-mortem samples (Chan et al. 1983). Phosphine will rapidly get absorbed through the airway tract or gastrointestinal mucosa. The remaining Aluminium sulphate [Al_2_ (SO_4_) _3_] may react with water to produce sulfuric acid inside our body (Munim).

$$ {\mathrm{Al}}_2{\left({\mathrm{SO}}_4\right)}_3 + 6\ {\mathrm{H}}_2\mathrm{O}\to 2\mathrm{A}\mathrm{l}{\left(\mathrm{OH}\right)}_3 + 3{\mathrm{H}}_2{\mathrm{SO}}_4 $$

It could be the likely reason for the reappearance of sulfuric acid like corrosion in oesophagus and stomach.

Histopathological changes in the gastrointestinal tract in acid poisoning ranges from simple hyperaemia/erosions to diffuse transmural necrosis (Contini & Scarpignato 2013). But in a case of phosphide poisoning, almost all vital organs are involved. Microscopic changes in phosphine poisoning in the liver are fatty change, necrosis and sinusoidal congestion (Aggrawal 2006). Kidney microscopy showed glomerulus congestion, intra-parenchymal congestion and tubular degeneration in phosphide poisoning (Mehrpour et al. 2008). We also observed diffuse acute tubular necrosis of kidney, sinusoidal congestion of the liver, and transmural necrosis of stomach wall on microscopy.

In case of metal phosphide poisoning, a forensic scientist generally carries out a phosphide/phosphine gas test for the detection and identification of phosphide in the viscera (Bhadkambekar et al. 2008). Silver nitrate test is used for the detection of phosphine after acid hydrolysis by sulfuric acid on metal phosphide. But this test will answer only for unutilized phosphides and not for absorbed phosphine. So there is no role in testing the blood for phosphine (Balali-Mood M. Phosphine. Simple Qualitative Test(s). Available at http://www.inchem.org/documents/pims/chemical/pim865.htm#DivisionTitle:8.2.1.1. Accessed 6 Nov 2015). If this test is positive, the presence of zinc or aluminium in the viscera at high levels could provide the information about the specific metallic phosphides. But, the human body also contains trace amount of these elements, within certain ranges, which can be differentiated from their quantities. Hence, it is ideal to detect and quantify these metals along with phosphide determination to be able to frame a definitive opinion (Bhadkambekar et al. 2008). But in developing countries like India, because of high case load and procedural cost, quantitative analysis is not routinely performed. To detect the aluminium, we should add ammonium chloride and ammonium hydroxide to the sample solution which will form a gelatinous white precipitate, and will turn into red lakes on addition of alcoholic alizarin (Vogel 1979a).

Dilute sulfuric acid is absorbed as hydrogen and sulphate ion through the mucous membrane. About 85–90% of inorganic sulphate is excreted as salts with sodium, potassium, calcium or ammonia (Biologic effects of exposure). Sulphate ion can be detected by the addition of 1% barium chloride and concentrated nitric acid to the sample solution, and a positive result will be indicated by formation of a white precipitate which will persist on heating or dilution (Vogel 1979b). From our history, clinical, laboratory and autopsy findings, the forensic scientist approached the case. But here, already he survived for 5 days and there may be a scant quantity of non-metabolized form of phosphine left. This could be the reason why phosphine or phosphide was not detected from any post-mortem samples.

Tha case history, clinical features, laboratory and autopsy findings, histopathology and chemical analysis reports were analysed to opine the cause of death as combined effect of pulmonary oedema and acute tubular necrosis of kidney as a result of aluminium phosphide and sulfuric acid poisoning.

## Conclusion

The autopsy findings due to mixing up of these two substances has not been reported in medical literature, and the typical post-mortem appearance of these individual compounds may change because of the chemical reactions occurring inside and outside the body. As far as the forensic scientists are concerned, accurate history and relevant findings should be perused to them for their proper guidance.
